# Cross-sectional changes in weight status and weight related behaviors among Australian children and Australian Indigenous children between 2010 and 2015

**DOI:** 10.1371/journal.pone.0211249

**Published:** 2019-07-09

**Authors:** Louise L. Hardy, Rona MacNiven, Tuguy Esgin, Seema Mihrshahi

**Affiliations:** 1 Prevention Research Collaboration, Sydney School of Public Health, Sydney Medical School, The University of Sydney, Sydney, New South Wales, Australia; 2 School of Public Health and Community Medicine, Faculty of Medicine, The University of New South Wales, Sydney, New South Wales, Australia; 3 Discipline of Exercise and Sport Science, Faculty of Health Sciences, The University of Sydney, Sydney, New South Wales, Australia; 4 School of Medical and Health Sciences, Edith Cowan University, Perth, Western Australia, Australia; Cincinnati Children's, UNITED STATES

## Abstract

**Background:**

Since 2006 there has been substantial long-term investment in school-based child obesity prevention programs in New South Wales (Australia). Whether these programs have led to population level improvements in children’s weight status and weight-related behaviors are yet to be determined. The purpose of this study was to describe changes in children’s weight status and weight-related behaviors, including Indigenous children, who are at greater risk of poorer health outcomes than non-Indigenous children.

**Methods:**

Representative cross-sectional population surveys conducted in 2010 and 2015 among children age 5–16 years (n = 15,613). Objective measurements included height, weight, waist circumference, cardiorespiratory fitness, and fundamental movement skills. Indigenous status and indicators of weight-related behavior (i.e., diet, physical activity, school travel, screen-time) were measured by questionnaire with parents responding for children age <10 years and self-report by children age ≥10 years.

**Results:**

The prevalences of overweight/obesity, obesity and abdominal obesity were higher in 2015, than 2010, and higher among Indigenous than non-Indigenous children at both timepoints. There were some small positive changes towards healthier weight-related behaviors between surveys among all children, but many unhealthy weight-related behaviors remain highly prevalent. The magnitude of changes and the 2015 prevalences of weight-related behaviors were generally similar for Indigenous and non-Indigenous children.

**Conclusions:**

Schools play an important role in health promotion, but our findings suggest the current approaches need re-thinking. Upstream factors that shape weight-related behaviors such as the regulation of the food industry and food environment, urban, neighborhood and public transport planning must be including in solutions to ensure populations can eat healthily and be physical active.

## Introduction

Since 2002 there has been substantial investment in New South Wales (NSW) Australia to reduce child obesity through a succession of state plans, policies, and programs to support the healthy development of children from 0–18 years.[1–3] The NSW government strategy was to encourage and support opportunities for the community to be healthy through the delivery of evidence-based, interactive, and relevant programs. Within the education sector, child obesity prevention has been addressed through a steady implementation of specific state level mandatory policies and recommended programs on healthy eating and physical activity including teacher resources and funding support in early childhood settings[4] and schools.[5–8]

Scaling-up school health programs is challenging, and the realization of health benefits of these programs may take many years. A recent study showed that there has been an increase in the proportion of schools adopting healthy eating and physical activity programs and policies between 2006 and 2013, however universal adoption across schools is yet to be achieved.[9] An important knowledge gap is whether these programs have achieved their objective to improve children’s healthy eating and physical activity leading to subsequent reduction in child obesity rates. In contrast to controlled small intervention studies, the rigorous evaluation of multiple scaled-up programs is often highly complex and not economically feasible. Rather, serial population health surveillance surveys can be used to provide estimates of prevalence, change and trends in health behaviors and health outcomes.

Between 1985 and 1995 the prevalence of obesity trebled and overweight doubled among Australian children,[10] however data collected in 2010 and 2015 indicates that the prevalence of overweight/obesity may have reached a plateau.[11] Between 2010 and 2015 the unadjusted prevalence of overweight/obesity of NSW children age 5–16 years did not significantly change (23.2% vs 24.5%, respectively)[12] which may suggest the net effect of all community and school-based investments in NSW, which started around 2006, may be influencing the incidence of overweight/obesity in children. While this is encouraging at a population level, the data also show there are clear and persistent socio-economic and cultural inequalities in the distribution of overweight/obesity in NSW children, and these disparities have increased over time.[11, 13]

Aboriginal and Torres Strait Islanders (i.e., Indigenous) are Australia’s First Nation people and are over-represented amongst disadvantaged groups, which has contributed to worse health outcomes and lower life expectancies than non-Indigenous populations.[14]. Aboriginal and Torres Strait Islanders are defined as people of Aboriginal or Torres Strait Islander descent who identify as Aboriginal or Torres Strait Islander and are accepted as such by the community in which they live.[15] In NSW 95% of Indigenous people are Aboriginal [16] however we will use the term Indigenous as it is inclusive of Torres Strait Islanders. Health inequalities arise from social inequality, and Indigenous people in Australia have lower levels of educational attainment, employment, and income compared with non-Indigenous Australians.[14] Indigenous people account for 3.0% of the total Australian population and recent estimates show that overweight/obesity is higher among Indigenous than non-Indigenous children.[17]

In 2011–12 the total cost of obesity in Australia was AUD$8.6 billion,[18] yet there are no financial data available on the total expenditure in NSW on the implementation and on-going costs of child obesity prevention programs implemented since 2006. Serial surveillance of children’s weight and weight related behavior is currently the best proxy measure to examine whether the overall investments by government and community in prevention have been effective. The purpose of this paper was to use representative survey data to describe changes in NSW children and in Indigenous children’s weight and weight-related behaviors between 2010 and 2015. We also report on the presence of school-based healthy eating and physical activity programs in NSW in 2015 and children’s exposure to these programs.

## Materials and methods

The data come from repeat, cross-sectional representative, population monitoring surveys of NSW school children age 5–16 years that were conducted between February and March in 2010 and 2015. The survey methodology, including sampling frame, sample size calculations, staff and indicator measurement protocols are standardized across survey years to allow comparability and to examine change in weight and weight-related behaviors over time, and are described in detail in the survey reports.[12, 19]

Briefly, the sampling frames comprise of primary and high schools randomly selected from each education sector (government, Catholic and independent) across socio-economic quintiles and in urban and rural areas, to provide a representative sample of NSW school-age children. The sampling frames were based on a two-stage probability sample (school, student) and comprised all NSW schools, except special education (e.g., blind, sport) and small (<180 enrolments) schools. Schools were randomly sampled from each education sector proportional to enrolment in that sector, and two classes randomly selected from each target year and children in those classes invited to participate. The surveys were school-based, and the data collected by trained teams of teachers. Ethics approvals were given by The University of Sydney, NSW Department of Education and Communities, the NSW Catholic Education Commission and the Aboriginal Health and Medical Research Human Ethics Committee. Written consent from parents was a requirement for participation.

The primary purpose of the surveys were to report on the change in rates of overweight/obesity and weight-related behaviors in children in relation to the NSW State Health Plan target (i.e., Reduce overweight/obesity rates of children age 5–16 years to 21% by 2015).[2] Cluster sampling was employed for the surveys to adjust for the correlation of measures for children in the same school. This required that the sample size be inflated to account for the clustering effect. The highest cell prevalence for obesity/overweight in 2010 was 29.9% and sample size was calculated using p1 = 0.30 and p2 = 0.20, which enabled detection of a difference of 10% in the prevalence between groups, with 80% power and alpha = 0.05. Post-stratification weights were calculated to permit inferences from children included in the sample to the populations from which they were drawn, and to have tabulations reflect estimates of population totals. Post-stratification weights were calculated using the NSW student population frame provided by the Australian Council for Educational Research.

### Measures

Height, weight, and waist circumference were measured by two field staff using standard procedures.[20] One staff member took the measurement, the other recorded the measurement. Height was measured to the nearest millimeter using the stretch stature method and a portable stadiometer (Mentone, model PE 187). Weight was measured to the nearest gram using Tanita portable scales (model HD380). Waist circumference was measure to the nearest millimeter, at the level of the narrowest point between the lower rib and iliac crest with a steel anthropometric tape (Lufkin W606PM). Height and weight were used to calculate body mass index (BMI kg/m^2^) and children categorized according to international pediatric age-sex adjusted cut-points as overweight/obese or obese.[21] Abdominal obesity, an index of cardio-metabolic risk, was determined by the waist-to-height ratio (WtHR, cm/cm) and children categorized as low risk (<0.5) or high risk (≥0.5).[22]

Parents of children in Kindergarten, grades 2 and 4 (i.e., age <10 years) completed a questionnaire which was distributed at the time of consent. Parents provided their child’s demographic information and on indicators of weight-related behaviors. Children in grades 6, 8, and 10 (i.e., age >10 years) completed the same questionnaire during the school visits. Demographics included sex, date of birth, postcode of residence, and Indigenous status. Postcode of residence was used as a proxy for socioeconomic status (SES), based on the Australian Bureau of Statistics’ Index of Relative Socio-economic Disadvantage (IRSD).[23] The IRSD is one of the socioeconomic indices for areas, which is updated after each Census. The IRSD is an ordinal measure (based on a standard score of 1000 with a standard deviation of 100). Postcode of residence was also used to define residence (urban or rural) using the Australian Statistical Geography Standard Volume 5; Remoteness Areas.[24] 2011 Census data were used for both surveys. Indigenous status was determined by asking *Are you of Aboriginal and/or Torres Strait Islander origin*? *(Response; Yes*, *No*, *Don’t know)*. Don’t know responses were recoded as No (1.9%).

Indicators of dietary intake were collected using a validated short food frequency questionnaire developed for population-based surveys.[25] Respondents reported the usual consumption of fruit, vegetables, energy-dense, nutrient poor foods (EDNP, i.e., fried potato products, salty snack foods, snack foods, confectionary, ice-cream). Children’s fruit and vegetable intakes were categorized according to the daily serves recommended by Australian Dietary Guidelines; fruit for children age 4–18 years was 2 serves, and vegetables for children age 4–7 years was 2 serves, children age 8–11 years was 3 serves, and children age 12–18 years was 5 serves.[26] The response categories for EDNP foods were never/rarely, 1–2 times/week, 3–4 times/week, 5–6 times/week, daily, and 2 or more times/day.

For the analysis, a junk food intake measure score was calculated from the combined consumption of EDNP foods and children’s scores ranked into tertiles.[27] Additional dietary behavior questions included frequency of eating breakfast (daily vs not daily), eating dinner in front of the TV (<5 vs ≥5 times/week), and how often good behavior was rewarded with sweets (never vs usually/sometimes). Recreational screen-time was measured by questionnaire[28] and children were categorized according to screen-time recommendation (<2 or ≥2hours/day).[29] Respondents also reported if there was a TV in the child’s bedroom (yes/no) and whether there were rules on screen-time (usually vs never/sometimes).

Cardiorespiratory fitness was assessed in children in grade 4 and above using the 20-meter shuttle run test.[30] For the analyses, the level and shuttle were converted to the total number of laps completed and children were categorized as ‘adequately fit’ or ‘unfit’ according to the age-sex adjusted FITNESSGRAM cut-points (S1 Table).[31] Seven fundamental movement skills (FMS) were assessed; four locomotor skills (sprint run, vertical jump, side gallop, leap) and three object control skills (catch, over arm throw, kick) using process-oriented checklists for each skill.[32] Children who demonstrated advanced skills in ≥2 object control and ≥3 locomotor skills were categorized as having advanced object control and locomotor skills, respectively.[33]

In 2015, the Principal of each participating primary school (n = 42) completed a School Environment Questionnaire, which included a question on the schools’ participation in established government primary school programs to address child obesity in NSW.[12] Responses for each school were linked to children’s data (via the school ID) to determine if these programs were present at their school.

### Analysis

We used Complex Samples SPSS (version 22 for Windows, IBM Corporation, Chicago, IL, USA) to account for post-stratification sampling weights and the cluster design (school sector and school) of the study and adjust for the standard errors and produce 95% confidence intervals. Alpha was set at .05. Missing data overall was 2.6% for anthropometry outcomes, 2.9–3.4% for dietary outcomes, 1.1–5.2% for physical activity outcomes, and 3.2–3.9% for screen-time outcomes. Pairwise deletion was used for missing data (i.e., missing data were not replaced). Generalized linear models (CSGLM) were used to generate prevalences with 95% confidence limits (95%CI) by survey year, adjusted for age, sex, SES (SEIFA score), and residence (urban, rural). Descriptive statistics were used to report the presence of government school-based programs in schools.

## Results

The characteristics of the children are presented in Table 1, for all children, non-Indigenous children, and Indigenous children by survey year. The survey response rates were 57% and 62% and Indigenous children comprised 4.1% and 3.9% of the sample in 2010 and 2015, respectively. Indigenous children were more likely to live in rural areas and have lower SEIFA scores than their non-Indigenous peers in both survey years.

**Table 1:** Characteristics of the sample by population groups and survey year.

	All children			Non-Indigenous			Indigenous*		
Survey year	2010	2015	P-value	2010	2015	P-value	2010	2015	P-value
n (%)	8,058	7,555	.77	7,594 (96.8%)	7,191 (96.6%)	.65	254 (4.1%)	251 (3.9%)	.77
Girls (%*; 95%CI)*)	47.9 (46.9, 48.9)	50.3 (46.9, 53.7)	.*17*	47.7 (46.6, 48.9)	50.6 (47.1, 54.1)	.12	55.8 (51.5, 59.9)	46.8 (40.3, 59.7)	.*03*
Age (years, *95%CI*)	10.4 (10.1, 10.8)	10.3 (9.7, 11.0)	.85	10.4 (10.1, 10.8)	10.3 (9.7, 11.0)	.79	10.7 (10.5, 11.0)	10.4 (9.5, 11.2)	.15
SES *(*mean, *95%CI*)**	986.3 (974.6, 998.0)	999.0 (984.1, 1014.0)	.19	989 (977.3, 999.9)	1000 (984.8 1015.1)	.24	941.4 (936.5, 946.3)	982.7 (968.4, 997.0)	< .001
*Residence (%; 95%CI)*									
Urban	84.1 (81.5, 86.5)	76.2 (65.4, 84.5)	.08	85.1 (82.0, 87.7,)	76.7 (65.9, 84.8)	.06	61.3 (59.9, 62.6)	65.1 (48.6, 78.6)	.63
Rural	15.9 (13.5, 18.5)	23.8 (15.5, 34.6)		14.9 (12.3, 18.0)	23.3 (15.2, 34.1)		38.7 (37.4, 40.1)	34.9 (21.4, 51.4)	

* Indigenous children; 2010 survey primary school n = 157, secondary school n = 97; 2015 survey primary school n = 176, secondary school n = 75.

** SES = Socioeconomic status, based on the Australian Bureau of Statistics’ Index of Relative Socioeconomic Disadvantage score (SEIFA score)

**Fig 1:**
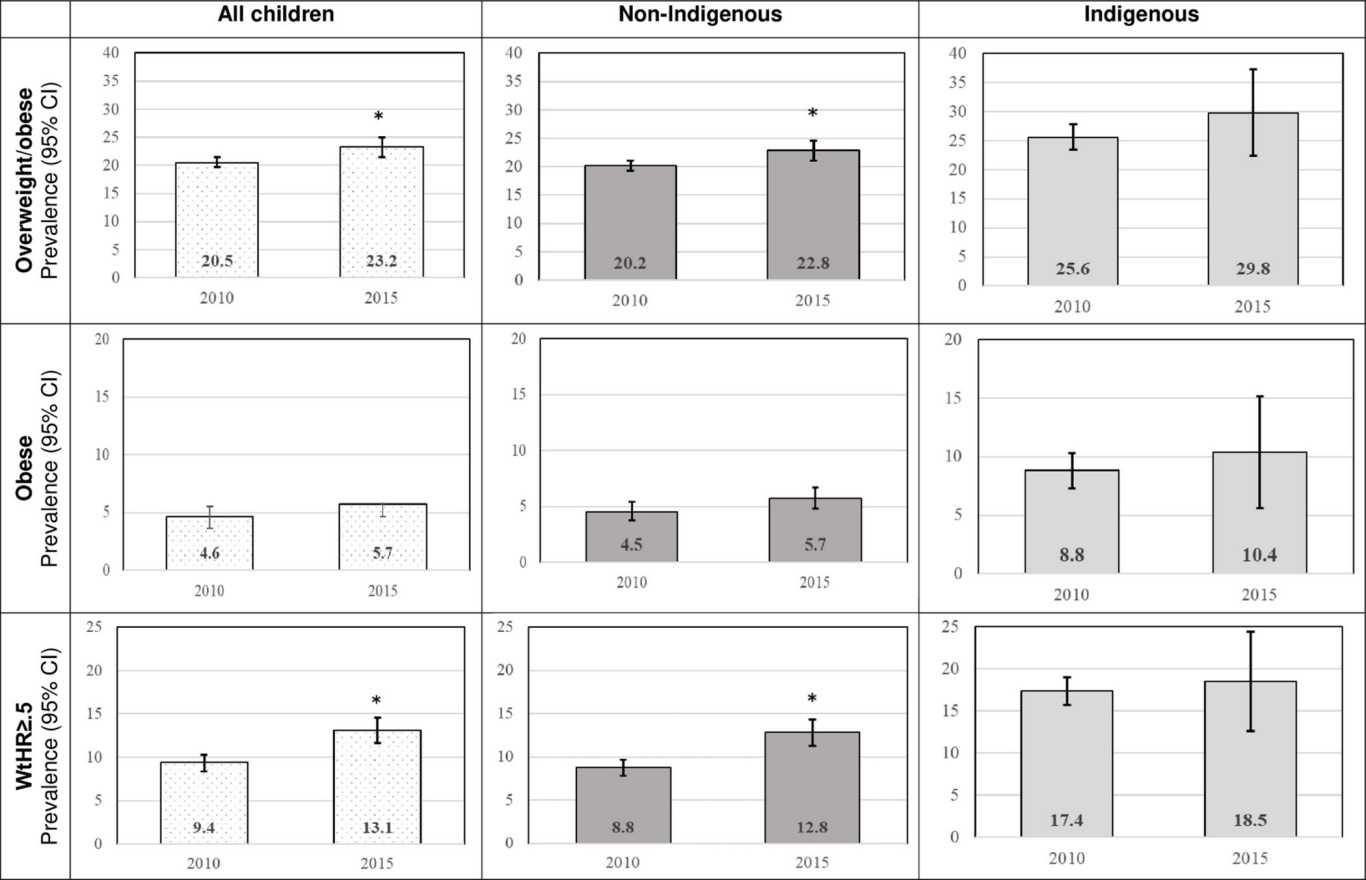
Adjusted prevalence (%, 95%CI) of children’s weight status in 2010 and 2015.

Fig 1 shows the adjusted prevalence of overweight/obesity, obesity and WtHR≥0.5 were higher in 2015 than 2010 for all children and each sub-population group, and the prevalences were higher among Indigenous than non-Indigenous children at both time points. In 2015, the prevalences of overweight/obesity and WtHR ≥0.5 were significantly higher than 2010, for all and non-Indigenous children. Although not statistically significant, the changes in the prevalences among Indigenous children show that each indicator of weight status were higher in 2015 than in 2010.

Table 2 shows the prevalence of weight-related behaviors in 2010 and 2015 by population group, adjusted for age, sex SES, and residence. While the changes in many behaviors for each child group were in the desired direction, most behaviors remained at sub-optimal levels. In 2015, indicators of dietary patterns and behaviors showed that only 7% of children ate the recommended daily serves of vegetables, 25–30% did not eat breakfast daily, approximately one in five had a high junk food intake and ate dinner in front of the TV ≥5/week, and almost half were rewarded with sweets for good behavior. Physical activity indicators showed that less than half of the children demonstrated advanced FMS, three in five had adequate fitness, one in seven used active travel to school, and two in five were driven to school. Approximately half the children met the recommended screen-time on school days and only one in five met the recommendation on weekend days. Approximately 30–40% of children had a TV in their bedroom, and more than half had no rules on screen-time. In contrast to 2010, many of the prevalences in 2015 were similar among child groups, but the prevalences for eating breakfast daily, meeting screen-time recommendations on a week day and having a TV in the bedroom were lower among Indigenous, than non-Indigenous children.

**Table 2:** Adjusted prevalence of weight-related behaviors by child group and survey year (%; 95%CI).

	All children		Non-Indigenous children		Indigenous children	
	2010 (n = 8,059)	2015 (n = 7,555)	2010 (n = 7,594)	2015 (n = 7,191)	2010 (n = 254)	2015 (n = 251)
*Food consumption*						
Meets fruit recommendation ^a^	73.9 (69.1, 78.7)	79.1 (76.9, 81.4)	74.5 (70.0, 78.9)	**79.3 (77.1, 81.5)**	63.3 (56.3, 70.4)	**79.5 (74.0, 85.0)**
Meets vegetable recommendation ^b^	5.4 (4.8, 6.0)	**7.5 (6.4, 8.5)**	5.4 (4.8, 5.9)	**7.5 (6.4, 8.5)**	6.2 (2.8, 9.5)	8.1 (4.3, 11.9)
Eat breakfast daily	80.2(76.4, 84.0)	76.1 (74.0, 78.2)	80.8 (77.2, 84.4)	76.8 (74.7, 78.9)	68.3 (61.0, 75.7)	68.4 (62.0, 74.7)
Junk Food Index Measure						
Low (score 0–5)	41.6 (39.6, 43.7)	**50.8 (48.5, 53.2)**	42.2 (40.1, 44.2)	**51.1 (48.7, 53.5)**	30.6 (22.7, 38.5)	**48.0 (40.2, 55.7)**
Middle (score 6–8)	33.1 (32.0, 34.2)	**28.6 (27.3, 29.9)**	33.2 (31.9, 34.5)	**28.7 (27.3, 30.0)**	32.3 (29.5, 35.1)	28.7 (23.3, 34.1)
High (score 9–25)	25.3 (23.6, 27.0)	**20.5 (18.6, 22.5)**	24.6 (23.2, 26.1)	**20.2 (18.4, 22.1)**	37.1 (31.5, 42.6)	23.3 (16.4, 30.2)
*Family practices*						
Eats dinner in front of TV <5/week	81.2 (78.0, 84.3)	83.4 (81.9, 84.9)	81.7 (79.0, 84.4)	83.6 (82.2, 85.1)	69.3 (61.2, 77.3)	81.7 (75.3, 88.1)
Parents never reward good behavior with sweets	48.1 (44.4, 51.8)	46.6 (44.5, 48.7)	48.7 (45.5, 51.8)	46.8 (44.8, 48.8)	38.6 (31.6, 45.5)	46.1 (37.8, 54.4)
*Physical activity outcomes*						
Advanced locomotor skills	35.9 (32.2, 39.7)	**50.0 (46.9, 53.0)**	36.6 (32.8, 40.3)	**50.3 (47.3, 53.3)**	26.8 (23.9, 29.7)	**45.7 (37.3, 54.1)**
Advanced object control skills	43.0 (42.0, 44.1)	**49.1 (46.7, 51.5)**	43.0 (41.8, 44.1)	**49.2 (46.7, 51.6)**	44.9 (41.3, 48.5)	50.0 (43.5, 56.6)
Healthy cardiorespiratory fitness zone	66.8 (64.7, 68.8)	**61.1 (57.7, 64.6)**	66.9 (64.9, 68.8)	**61.3 (57.8, 64.8)**	63.4 (54.0, 72.7)	58.1 (49.3, 67.0)
*School travel*						
Active travel to school	17.9 (9.8, 26.0)	15.8 (13.5, 18.2)	17.4 (9.7, 25.1)	15.5 (13.1, 17.9)	22.9 (10.8, 35.12)	20.8 (13.4, 28.3)
Driven to school	31.2 (23.2, 39.2)	39.8 (36.3, 43.3)	32.0 (24.1, 39.9)	40.1 (36.5, 43.6)	20.7 (14.8, 26.7)	**35.8 (27.8, 43.8)**
*Screen-time*						
Meets recommendation on weekdays	55.3 (50.7, 60.0)	54.2 (51.4, 56.9)	56.5 (52.2, 60.7)	54.8 (52.0, 57.6)	32.2 (28.6, 35.9)	**46.3 (39.7, 52.8)**
Meets recommendation on weekends	19.1 (18.0, 20.3)	20.3 (18.8, 22.1)	19.1 (17.8, 20.5)	20.3 (18.7, 22.0)	16.7 (14.3, 19.1)	19.6 (12.6, 26.6)
No TV in the bedroom	68.2 (60.6, 75.9)	76.9 (74.6, 79.1)	69.5 (62.1, 76.9)	**78.1 (76.0, 80.2)**	43.7 (37.6, 49.8)	53.3 (44.2, 62.4)
Usually has rules on screen-time	49.7 (46.3, 53.0)	46.3 (44.3, 48.2)	50.1 (46.3, 53.8)	**46.3 (44.3, 48.3)**	41.3 (38.5, 44.2)	46.8 (36.6, 57.0)

Bold values represent significant difference between 2010 and 2015 within each child group. Models adjusted for sex, age, residence (rural/urban), and socioeconomic status (SEIFA score).

^a^ Daily fruit recommendations for children age 4–18 years was 2 serves:

^b^ Daily vegetable recommendations for children age 4–7 years was 2 serves and children age 8–11 years was 3 serves, children age 12–18 years was 5 serves. [26]

The presence of government primary school healthy eating and physical activity programs reported by the Principals are presented in S2 Table. Several factors need to be considered in the interpretation of the findings. In 2015, 42 primary schools participated in the survey, which represented approximately 3% of NSW primary schools, and the Principals only reported whether the program was operating in the school. We did not collect information on the duration that the program had been in place, which children participated in the programs, or program fidelity.

There were no statistical differences between Indigenous and non-Indigenous children’s exposure to these programs, however there were differences in program presence in schools. Nine healthy eating and physical activity programs were measured in primary school, with schools averaging 4.8 (SD 2.1) programs. Five percent of schools had one program and 5% of schools reported all nine programs were present (S3 Table). The most prevalent programs in primary schools were fruit/vegetable/water programs, and the least prevalent were school kitchen gardens.

## Discussion

The purpose of this study was to describe changes in NSW children’s weight status and weight-related behaviors following more than a decade of multiple, sustained, evidence-based, scaled-up child obesity prevention programs. Given the health gap between Australia’s Indigenous and non-Indigenous populations we also present the prevalence for Indigenous children to identify lifestyle behaviors that may need attention. Our findings indicated the adjusted prevalence of overweight/obesity, obesity and WtHR≥0.5 have increased between 2010 and 2015, among all children. The pattern and magnitude of change in weight status and weight-related behaviors in Indigenous children were similar to non-Indigenous children, but the prevalences were consistently higher among Indigenous children in 2010 and 2015.

A limitation of this study was the small sample size for Indigenous children. The survey sampling frames were designed to represent NSW children age 5–16, not Indigenous children, however Indigenous children represented ~4% of the sample survey which was higher than the NSW prevalence (2.9%).[34] While small sample size precludes determining statistically significant differences, statistical significance simply addresses whether to accept or reject a null or directional hypothesis, without providing information on the magnitude or direction of the difference. Given the social and health disadvantage of Indigenous children we believe it is important for policy and for evidence-based practice to report on the epidemiology of their weight and weight-related behaviors, and to assess whether these have improved over time.

There were some small improvements in weight-related behaviors of all children, however the prevalence of most of these behaviors remain sub-optimal, potentially contributing to the increase in children’s weight status. Similar findings that NSW rural dwelling Indigenous and non-Indigenous children’s physical activity, including school travel have declined and are at low levels.[35] Children’s dietary behaviors remain a concern, especially the consumption of vegetables with only one in sixteen children in this study meeting the recommended daily serves. The health benefits of eating vegetables (and fruit) have long been recognized and are prominent in dietary guidelines, with the Australian government in 2005 recommending all primary schools implement fruit and vegetable programs.[36] The majority of primary school children in this study attended schools with fruit and vegetable programs and while there seemed to be gains in children’s intake of fruit, these programs overall have had only marginal returns in the long term. This indicates different strategies are required to improve children’s fruit and vegetable intakes. Access, affordability, and availability of fruit and vegetables may be difficult for some groups, including Indigenous people[26] however other factors must also be influencing dietary patterns and behaviors.

We found that almost one quarter of children had a high junk food intake, which is of critical concern. Solutions require up-stream policies such as urban planning to curb the growth of food swamps (i.e., suburbs with a higher ratio of fast food retailers and convenience stores (unhealthy) to grocery stores, supermarkets and farmers markets (healthy)) [37] especially around schools, and restrictions on marketing and advertising to children, which may help to influence children’s food environments. [38]. Similarly, parents are the nutritional gatekeepers of children and efforts to improve children’s diets must involve parents. In this study, indicators of the home food environment for many children were not ideal. More than half of the parents reward their child’s good behavior with sweets, a practice that not only increase caloric intake but can shape future eating habits.[39] Around one in five children ate dinner in front of the TV three times a week, which is another practice associated with poor diet quality due to exposure to the advertising of highly processed foods and soft drinks.[40] Too many children in this study did not eat breakfast daily, with almost one third of Indigenous children missing this meal. The association between skipping breakfast and overweight is contentious,[41] however there is evidence that children who skip breakfast tend to have poorer diet quality.[42]

While we did not measure food insecurity, this has been identified as a leading health issue in Indigenous communities and is associated with consuming foods of poor quality because they are the lowest cost options.[43] A recent review identified that effective nutrition programs in Indigenous communities require community engagement during the development and implementation to ensure community needs and priorities are addressed.[44] Breakfast programs in low income communities could have merit, but other social and cultural determinants such as gaps in knowledge about healthy choices, budgeting and cooking skills, also must be addressed to improve all children’s dietary intakes.

Live Life Well @ School is a flagship government primary school physical activity program operating since 2008 with a focus on FMS,[45] yet indicators of children’s physical activity and FMS continue at sub-optimal levels. Our findings reflect the D- awarded to Australia by the Active Health Kids Global Alliance, with Australian children’s participation in physical activity rated 32^nd^ out of 43 countries internationally.[46] There were significant improvements in children’s FMS, particularly locomotor skills, but less than half of the children had advanced FMS. FMS are the building blocks of physical activity[47] and the low prevalence of advanced skills may be a contributing factor to the low levels of children’s cardiorespiratory fitness. Only three in five children demonstrated adequate cardiorespiratory fitness with declines occurring between 2010 and 2015. Cardiorespiratory fitness is a marker of cardiovascular health and the current levels indicate the importance to promote children’s physical activity levels to protect them from the risk of future cardiovascular diseases. Skill development is pivotal as the association between FMS and physical activity is reciprocal; more physical activity increases skills, and greater skills increase physical activity which contributes to cardiorespiratory fitness.[48]

Parents also need to be included in promoting children’s physical activity. Active travel to school potentially contributes to children’s daily physical activity,[49] yet only one in seven children in this study use active transport to get to school, and proportion of children been driven school rose significantly between 2010 and 2015. Reasons for the increase in being driven to school are not clear, but other Australian research suggests factors associated with children not using active transport to/from school include proximity to school, the safety of the route, and family time constraints.[50] Recent Australian studies have reported that many children did not attend the school closest to them because their parents can choose alternatively located schools elsewhere, and that increase in chauffeuring children particularly to school, has led to declines in children’s independent mobility.[51, 52]

The most popular recreational sedentary behavior in children is screen-time, and the Australian guidelines recommend children age 5–18 years limit screen-time to less than 2 hours/day.[29] We measured children’s recreation screen-time (TV, DVD, e-games, computer use smart phones) outside of school hours and found approximately half the children meet this recommendation on weekdays and only one in five on weekend days. Screen technologies are embedded in most aspects of daily life and the education sector needs to ensure children are digitally savvy and prepared for future workforces. While this does raise the question of whether current screen-time recommendations are realistic, the evidence consistently demonstrates that excessive screen-time during childhood is associated with increased risk of chronic disease and unfavorable child development outcomes.[53]

Ecological models show that the home environment determines child behavior, and homes with a screen-time promoting environment (e.g., TVs in bedrooms, no rules on screen-time, parents’ screen-time) are associated with increased screen-time. [54] There were differences in the proportion and change between surveys of TVs in children’s bedrooms between child groups, with a larger proportion of non-Indigenous than Indigenous children reporting there were no TVs in their bedrooms. Almost four in five children did not have a TV in the bedroom, however we did not ask about other screen devises which may have replaced TVs, such as tablets and computers. Less than half of the children’s parents usually had screen-time rules, the prevalence was lower in 2015 than in 2010, however in contrast, the prevalence among Indigenous children in 2015 was higher than in 2010. We did not measure parents’ screen-time, other research shows that few parents limit their own screen-time, which may be a factor in parental decisions to set screen-time rules.[55]

In summary, there has been long-term investment in child obesity prevention programs, including state-wide school-based programs in NSW which has resulted in only small changes in some indicators of children’s weight-related behaviors. The higher rates of overweight/obesity and abdominal obesity in 2015, compared with 2010, indicate the current approaches to child obesity prevention are not achieving the desired outcomes. It is not clear why more than 10 years investment has had little or no impact on most weight-related behaviors, with the exception of fruit consumption. Potentially, because most primary schools have fruit/vegetable programs, school-based programs may have value added to the favorable increase in fruit consumption. Although we only asked about the presence of programs in 2015 our findings are consistent with other studies that suggest additional and/or different dissemination strategies are required to facilitate a greater adoption of policies and practice within schools.[9, 56] Importantly, there is no information on the fidelity or the quality of these school-based programs. Schools play an important role in health promotion, but our findings suggest the current approaches need re-thinking. Parents should be included in changing children’s (and their own) weight-related behaviors and government actions that address the food industry’s control of the food environment may be required to shift population behavior.

## Conclusions

In NSW, long-term government investment in child obesity prevention initiatives, including school-based programs have not changed children’s weight-related behaviors. Many unhealthy weight-related behaviors remain highly prevalent among NSW children. Reducing overweight/obesity prevalence in children is contingent upon tackling the drivers of obesity to enable the adoption of healthier weight-related behaviors.

## Supporting information

S1 Table FITNESSGRAM standards for 20-meter shuttle run test. (DOCX)

S2 Table Prevalence of programs in primary schools and children’s exposure to programs, by child group. (DOCX)

S3 Table Proportion of children attending primary schools in 2015 with government school-based child obesity prevention initiatives, (reported by the Principal). (DOCX)

## References

[1] NSW Health. NSW Childhood Obesity Summit Draft Program,. In: NSW Child Obesity Summit Committee, editor. Sydney: NSW Department of Health; 2002.

[2] NSW Ministry of Health. NSW Healthy Eating and Active Living Strategy: Preventing overweight and obesity in New South Wales 2013–2018. Sydney: NSW Ministry of Health; 2013.

[3] NSW Department of Health. NSW Government Plan for Preventing Overweight and Obesity in Children, Young People & their Families 2009–2011. Sydney: NSW Department of Health 2009.

[4] NSW Office of Preventive Health. Munch & Move 2008 [Accessed 7/11/2018]; Available from: http://www.preventivehealth.net.au/munch—move.html.

[5] NSW Department of Health, NSW Department of Education and Training. Fresh Tastes @ School NSW Healthy Canteen Strategy Canteen Menu Planning Guide 2006 [Accessed 7/11/2018]; Available from: https://healthy-kids.com.au/school-canteens/canteen-guidelines/nsw-healthy-school-canteen-strategy/.

[6] Healthy Kids Association. Crunch&Sip 2007 [Accessed 7/11/2018]; Available from: https://healthy-kids.com.au/teachers/crunch-sip/.

[7] NSW Office of Preventive Health. Live Life Well @ School 2008 [Accessed 7/11/2018]; Available from: http://www.preventivehealth.net.au/live-life-well—school.html.

[8] NSW Office of Preventive Health. Healthy Children's Initiative 2012 [Accessed 7/11/2018]; Available from: http://www.preventivehealth.net.au/.

[9] Nathan N, Wolfenden L, Williams CM, Yoong SL, Lecathelinais C, Bell AC, et al Adoption of obesity prevention policies and practices by Australian primary schools: 2006 to 2013. *Health Educ Res* 2015;30:262–71. 10.1093/her/cyu068 25516479

[10] Magarey AM, Daniels LA, Boulton TJC. Prevalence of overweight and obesity in Australian children and adolescents: reassessment of 1985 and 1995 data against new standard international definitions. *Med J Aust* 2001;174:561–4. 11453327 10.5694/j.1326-5377.2001.tb143435.x

[11] Hardy L, Mihrshahi S, Gale J, Drayton B, Bauman A, Mitchell J. 30-year trends in overweight, obesity and waist-to-height ratio by socioeconomic status in Australian children, 1985 to 2015. *Int J Obes (Lond)* 2017;41:76. 27847388 10.1038/ijo.2016.204 PMC5220161

[12] Hardy L.L., Mihrshahi S, Drayton BA, Bauman A. NSW Schools Physical Activity and Nutrition Survey (SPANS) 2015: Full Report. Sydney,: NSW Ministry of Health; 2017.

[13] Hardy LL, Jin K, Mihrshahi S, Ding D. Trends in overweight, obesity, and waist-to-height ratio among Australian children from linguistically diverse backgrounds, 1997 to 2015. *Int J Obes* 2019;43:116. 10.1038/s41366-018-0139-5 PMC6331387 29980760

[14] Australian Institute of Health and Welfare. The health and welfare of Australia’s Aboriginal and Torres Strait Islander peoples 2015 (Cat. no. 47). Canberra: AIHW; 2015.

[15] The Australian Institute of Aboriginal and Torres Strait Islander Studies. Indigenous Australians: Aboriginal and Torres Strait Islander people 2019 [Accessed 01/06/2019]; Available from: https://aiatsis.gov.au/explore/articles/indigenous-australians-aboriginal-and-torres-strait-islander-people.

[16] Australian Bureau of Statistics. Experimental Estimates and Projections, Aboriginal and Torres Strait Islander Australians, 1991 to 2021. In: Australian Bureau of Statistics, editor. Canberra, 2009.

[17] Australian Bureau of Statistics. Australian Aboriginal and Torres Strait Islander Health Survey: First Results, Australia, 2012–13 (Cat. no. 4727.0.55.001). Canberra: Australian Bureau of Statistics; 2013.

[18] Price Waterhouse Coopers. Weighing the cost of obesity: A case for action. Sydney: PwC; 2015.

[19] Hardy LL, King L, Espinel P, Cosgrove C, Bauman A. NSW Schools Physical Activity and Nutrition Survey (SPANS) 2010: Full Report. Sydney: NSW Ministry of Health; 2011.

[20] Olds T, Norton K, Commission AS. Anthropometrica: a textbook of body measurement for sports and health courses Sydney, Australia: UNSW Press; 1996.

[21] Cole TJ, Lobstein T. Extended international (IOTF) body mass index cut-offs for thinness, overweight and obesity. *Pediatr Obes* 2012;7:284–94. 10.1111/j.2047-6310.2012.00064.x 22715120

[22] Brambilla P, Bedogni G, Heo M, Pietrobelli A. Waist circumference-to-height ratio predicts adiposity better than body mass index in children and adolescents. *Int J Obes* 2013;37:943–6. 10.1038/ijo.2013.32 23478429

[23] Australian Bureau of Statistics. Census of Population and Housing: Socio-Economic Indexes for Areas (SEIFA), Australia—Data only, 2011 (Cat.no. 2033.0.55.001). -. Canberra: Australian Bureau of Statistics; 2013.

[24] Australian Bureau of Statistics. Australian Statistical Geography Standard (ASGS)- Remoteness Structure, (Cat. no. 1270.0.55.005). Canberra: Australian Bureau of Statistics; 2013.

[25] Flood V, K W, Rangan AM. Recommendations for short questions to assess food consumption in children for the NSW Health Surveys Sydney: NSW Centre for Public Health Nutrition; 2005.

[26] National Health and Medical Research Council. Australian Dietary Guidelines. Canberra: National Health and Medical Research Council; 2013.

[27] Boylan S, Hardy LL, Drayton BA, Grunseit A, Mihrshahi S. Assessing junk food consumption among Australian children: trends and associated characteristics from a cross-sectional study. *BMC Public Health* 2017;17:1–9. 10.1186/s12889-016-3954-4 28381213 PMC5382385

[28] Hardy LL, Booth ML, Okely AD. The reliability of the Adolescent Sedentary Activity Questionnaire (ASAQ). *Prev Med* 2007;45:71–4. 10.1016/j.ypmed.2007.03.014 17532371

[29] Department of Health. Australia's Physical Activity and Sedentary Behaviour Guidelines for Children (5–12 years) and Adolescents (13–17 years) Canberra: Commonwealth of Australia; 2014; Available from: http://www.health.gov.au/internet/main/publishing.nsf/content/health-pubhlth-strateg-phys-act-guidelines.

[30] Leger LA, Lambert J. A maximal multistage 20-m shuttle run test to predict VO2 max. *Eur J Appl Physiol Occup Physiol* 1982;49:1–12. 7201922 10.1007/BF00428958

[31] Welk GJ, Meredith MD. Fitnessgram/Activitygram Reference Guide. 2nd ed Dallas Tx: The Cooper Institute; 2008.

[32] NSW Department of Education and Training. Get skilled: Get active A K-6 resource to support the teaching of fundamental movement skills. Ryde: NSW Department of Education and Training; 2000.

[33] Booth ML, Okely T, McLellan L, Phongsavan P, Macaskill P, Patterson J, et al Mastery of fundamental motor skills among New South Wales school students: prevalence and sociodemographic distribution. *J Sci Med Sport* 1999;2:93–105. 10476973 10.1016/s1440-2440(99)80189-3

[34] Estimates of Aboriginal and Torres Strait Islander Australians, June 2011 [Internet]. Australian Bureau of Statistics. 2013. Available from: http://www.abs.gov.au/AUSSTATS/abs@.nsf/DetailsPage/3238.0.55.001June%202011?OpenDocument.

[35] Macniven R, Richards J, Turner N, Blunden S, Bauman A, Wiggers J., et al Understanding physical activity levels among Indigenous and non-Indigenous young people. *Rural Remote Health* 2019. 10.22605/RRH4876 31466453

[36] Australian Government Department of Health and Ageing. Evaluation of the National Go for 2&5 Campaign 2007 [Accessed Accessed 30/01/2018]; Available from: http://www.health.gov.au/internet/healthyactive/publishing.nsf/Content/2&5-eval-jan07.

[37] Cooksey-Stowers K, Schwartz M, Brownell K. Food swamps predict obesity rates better than food deserts in the United States. *Int J Environ Res Public Health* 2017;14:1366. 10.3390/ijerph14111366 PMC5708005 29135909

[38] Hawkes C, Smith TG, Jewell J, Wardle J, Hammond RA, Friel S, et al Smart food policies for obesity prevention. *The Lancet* 2015;385:2410–21. 10.1016/S0140-6736(14)61745-1 25703109

[39] Shan LC, McCafferty C, Tatlow-Golden M, O'Rourke C, Mooney R, Livingstone MBE, et al Is it still a real treat? Adults' treat provision to children. *Appetite* 2018;130:228–35. 10.1016/j.appet.2018.08.022 30118786

[40] Vik FN, Bjørnarå HB, Øverby NC, Lien N, Androutsos O, Maes L, et al Associations between eating meals, watching TV while eating meals and weight status among children, ages 10–12 years in eight European countries: the ENERGY cross-sectional study. *International Journal of Behavioral Nutrition and Physical Activity* 2013;10:58 10.1186/1479-5868-10-58 23675988 PMC3663732

[41] Blondin SA, Anzman-Frasca S, Djang HC, Economos CD. Breakfast consumption and adiposity among children and adolescents: an updated review of the literature. *Pediatr Obes* 2016;11:333–48. 10.1111/ijpo.12082 26842913

[42] Ramsay SA, Bloch TD, Marriage B, Shriver LH, Spees CK, Taylor CA. Skipping breakfast is associated with lower diet quality in young US children. *Eur J Clin Nutr* 2018;72:548–56. 10.1038/s41430-018-0084-3 29367733

[43] Browne J, Laurence S, Thorpe S. Acting on food insecurity in urban Aboriginal and Torres Strait Islander communities. Perth: Australian Indigenous HealthInfoNet; 2009 [Accessed 11/11/2018]; Available from: http://www.healthinfonet.ecu.edu.au/uploads/resources/17213_17213_acting-on-food-insecurity-in-urban-atsi-communities.pdf.

[44] Browne J, Adams K, Atkinson P, Gleeson D, Hayes R. Food and nutrition programs for Aboriginal and Torres Strait Islander Australians: an overview of systematic reviews. *Aust Health Rev* 2017. 10.1071/AH17082 28923162

[45] Bravo A, Innes-Hughes C, O’Hara BJ, McGill B, Rissel C. Live Life Well @ School: evidence and evaluation summary 2008–2013. Sydney: NSW Ministry of Health;; 2015.

[46] Active Healthy Kids Australia. Muscular Fitness: It’s Time for a Jump Start. The 2018 Active Healthy Kids Australia Report Card on Physical Activity for Children and Young People. Adelaide, South Australia,; 2018.

[47] Lubans DR, Morgan PJ, Cliff DP, Barnett LM, Okely AD. Fundamental movement skills in children and adolescents. *Sports Med* 2010;40:1019–35. 10.2165/11536850-000000000-00000 21058749

[48] Stodden DF, Goodway JD, Langendorfer SJ, Roberton MA, Rudisill ME, Garcia C, et al A Developmental Perspective on the Role of Motor Skill Competence in Physical Activity: An Emergent Relationship. *Quest* 2008;60:290–306.

[49] Martin A, Kelly P, Boyle J, Corlett F, Reilly JJ. Contribution of walking to school to individual and population moderate-vigorous intensity physical activity: systematic review and meta-analysis. *Pediatr Exerc Sci* 2016;28:353–63. 10.1123/pes.2015-0207 26882871

[50] Trapp GS, Giles-Corti B, Christian HE, Bulsara M, Timperio AF, McCormack GR, et al Increasing children’s physical activity: Individual, social, and environmental factors associated with walking to and from school. *Health Educ Behav* 2012;39:172–82. 10.1177/1090198111423272 21990572

[51] Stone J, Taylor E, Cole A, Kirk Y. ACOLA Securing Australia’s Future. Sustainable Urban Mobility Social Study: Barriers and pathways to sustainable urban mobility in Australia. 2014.

[52] Carver A, Timperio A, Crawford D. Parental chauffeurs: what drives their transport choice? *Journal of Transport Geography* 2013;26:72–7.

[53] Sigman A. Time for a view on screen time. *Arch Dis Child* 2012;97:935–42. 10.1136/archdischild-2012-302196 23044213

[54] Maitland C, Stratton G, Foster S, Braham R, Rosenberg M. A place for play? The influence of the home physical environment on children’s physical activity and sedentary behaviour. *International Journal of Behavioral Nutrition and Physical Activity* 2013;10:99 10.1186/1479-5868-10-99 23958282 PMC3765081

[55] Schoeppe S, Rebar AL, Short CE, Alley S, Van Lippevelde W, Vandelanotte C. How is adults’ screen time behaviour influencing their views on screen time restrictions for children? A cross-sectional study. *BMC Public Health* 2016;16:201 10.1186/s12889-016-2789-3 26932822 PMC4774016

[56] Hector D, Edwards S, Gale J, Ryan H. Achieving equity in Crunch&Sip: a pilot intervention of supplementary free fruit and vegetables in NSW classrooms. *Health Promot J Austr* 2017:-. 10.1071/HE16095 29248048

