# Supplementary material for: Cross-sectional changes in weight status and weight related behaviors among Australian children and Australian Indigenous children between 2010 and 2015
Source: PLoS One. 2019 Jul 9;14(7):e0211249. doi: 10.1371/journal.pone.0211249 (PMC6615594; doi:10.1371/journal.pone.0211249)
Supplement: S1 Table — (DOCX) [file pone.0211249.s001.docx]

**S1 Table** FITNESSGRAM standards for 20-meter shuttle run test.

|  | Minimum # laps to be classified adequately fit | |
| --- | --- | --- |
| Age (years) | Boys | Girls |
| 10 | 23 | 15 |
| 11 | 23 | 15 |
| 12 | 32 | 23 |
| 13 | 41 | 23 |
| 14 | 41 | 23 |
| 15 | 51 | 23 |
| 16 | 61 | 32 |
