# Supplementary material for: Cross-sectional changes in weight status and weight related behaviors among Australian children and Australian Indigenous children between 2010 and 2015
Source: PLoS One. 2019 Jul 9;14(7):e0211249. doi: 10.1371/journal.pone.0211249 (PMC6615594; doi:10.1371/journal.pone.0211249)
Supplement: S2 Table — (DOCX) [file pone.0211249.s002.docx]

**S2 Table** Prevalence of programs in primary schools and children’s exposure to programs, by child group.

| **Programs in schools (n)** | **Proportion with programs (%)** | **Children (%)** | | |
| --- | --- | --- | --- | --- |
|  |  | **All children** | **Non-Indigenous** | **Indigenous** |
| 1 | 5.0 | 4.3 | 4.2 | 7.8 |
| 2 | 12.5 | 13.7 | 13.6 | 7.8 |
| 3 | 7.5 | 5.2 | 5.3 | 3.6 |
| 4 | 17.5 | 18.8 | 18.8 | 19.8 |
| 5 | 22.5 | 22.5 | 22.7 | 19.8 |
| 6 | 12.5 | 12.3 | 12.2 | 15.0 |
| 7 | 12.5 | 11.6 | 11.3 | 17.4 |
| 8 | 5.0 | 5.2 | 5.3 | 4.8 |
| 9 | 5.0 | 6.4 | 6.5 | 4.2 |
