# Supplementary material for: Cross-sectional changes in weight status and weight related behaviors among Australian children and Australian Indigenous children between 2010 and 2015
Source: PLoS One. 2019 Jul 9;14(7):e0211249. doi: 10.1371/journal.pone.0211249 (PMC6615594; doi:10.1371/journal.pone.0211249)
Supplement: S3 Table — (DOCX) [file pone.0211249.s003.docx]

**S3 Table** Proportion of children attending primary schools in 2015 with government school-based child obesity prevention initiatives, (reported by the Principal)

| **NSW government primary school child obesity initiatives** | **Schools participating (n=40)** | **Children** | | |
| --- | --- | --- | --- | --- |
|  |  | **All children**  **(%, 95%CI)** | **Non-Indigenous**  **(%, 95%CI)** | **Indigenous**  **(%, 95%CI)** |
| *Primary school children (n)* |  | 5,145 | 4,969 | 176 |
| Fresh Tastes @ school* | 21 | 45.0 (29.7, 61.4) | 45.2 (30.2, 61.0) | 44.7 (24.5, 66.9) |
| Fruit, vegetable, or water breaks | 41 | 94.9 (81.0, 98.8) | 94.8 (80.7, 98.8) | 96.4 (85.3, 99.2) |
| Crunch and Sip** | 27 | 60.5 (44.5, 74.4) | 60.4 (44.4, 74.5) | 63.5 (39.0, 82.6) |
| School kitchen garden | 9 | 42.4 (27.3, 59.1) | 43.3 (28.0, 59.9) | 34.5 (16.7, 57.9) |
| Heart Foundation Jump Rope for Heart | 15 | 32.1 (19.0, 48.8) | 31.9 (7.5, 18.9) | 33.9 (10.7, 16.2) |
| NSW Premier's Sporting Challenge | 22 | 51.2 (39.3, 63.0) | 51.5 (39.6, 63.3) | 45.9 (26.2, 66.9) |
| Live Life Well @ School initiatives (child obesity prevention program) | 27 | 57.6 (41.4, 72.4) | 57.8 (41.5, 72.6) | 53.9 (30.1, 76.0) |
| Active After Schools Community | 18 | 37.6 (23.9, 53.7) | 38.0 (24.2, 54.2) | 33.6 (16.6, 56.2) |
| Outside of School Hours (OOSH) programs | 27 | 63.1 (46.6, 77.0) | 63.7 (47.3, 77.5) | 51.2 (28.2, 73.7) |

* All P>.05 (no difference between groups); * Fresh Tastes @ school is a health canteen program; **Crunch and Sip is a registered fruit and water program for schools which provides information and resources including materials for children to take home to their families.
